# Palbociclib: A New Option for Front-Line Treatment of Metastatic, Hormone Receptor–Positive, HER2-Negative Breast Cancer

**Published:** 2015-11-01

**Authors:** Harmony J. Bowles, Kathryn L. Clarke

**Affiliations:** University of Arizona Cancer Center, Tucson, Arizona

Breast cancer is the second leading cause of cancer-related death in American women. However, mortality has been steadily decreasing due to improvements in early detection and advances in treatment (American Cancer Society, 2015). Clinicians consider tumor histology along with the extent of disease and patient characteristics such as age, menopausal status, and comorbidities to personalize treatment options (National Comprehensive Cancer Network [NCCN], 2015). Depending on the biology and extent of disease, therapy can be multimodal and may include surgery, radiation, cytotoxic chemotherapy, endocrine therapy, or biologic therapies.

Breast cancer is frequently classified by receptor status: hormone receptor–positive (HR+), amplified expression of HER2/neu (HER2+), or triple-negative (no hormone receptors, nonamplified HER2) disease (Perou et al., 2000). For metastatic HR+ disease, the addition of targeted agents to antihormonal agents has shown significant clinical benefit (Baselga et al., 2012). Palbociclib (Ibrance) is the first drug of its class. It received accelerated approval from the US Food and Drug Administration (FDA), in combination with the aromatase inhibitor (AI) letrozole, for the treatment of postmenopausal women with estrogen receptor–positive (ER+), HER2-negative (HER2–) metastatic breast cancer (MBC) as initial endocrine-based therapy for metastatic disease (Pfizer Inc., 2015).

## PHARMACOLOGY AND MECHANISM OF ACTION

Palbociclib is an oral reversible inhibitor of cyclin-dependent kinases 4 and 6 (CDK4/6). CDK4/6 and cyclin D play a key role in progression from G1 to S phase of the cell cycle through phosphorylation of the retinoblastoma protein (Rb), which inactivates Rb function as a tumor suppressor (Choi & Anders, 2014; Roberts et al., 2012). Palbociclib, also known as PD-0332991, was developed from a group of pyridopyrimidine compounds after demonstrating potent and specific inhibition of CDK4/6, with greatest sensitivity to this inhibition observed in ER+ breast cancer cell lines (Fry et al., 2004; Finn et al., 2009). The specificity of palbociclib allows relatively specific inhibition of cancer cells while sparing many normal tissues, which are in a noncycling state (Dean, Thangavel, McClendon, Reed, & Knudsen, 2010). This improved its hematologic toxicity profile compared with earlier generations of CDK4/6 inhibitors (Finn et al., 2009).

Tumor cells must contain Rb if CDK4/6 inhibition is to be effective, and the majority of HR+ tumors have intact Rb gene expression (Trere et al., 2009). Additionally, in vitro treatment of breast cancer cell lines with the combination of palbociclib and antiestrogens leads to improved growth arrest compared with treatment with each drug alone (Finn et al., 2009). These observations informed the design of in vivo trials, as described here.

## CLINICALLY PERTINENT STUDIES

The current FDA indication for palbociclib was based on early results from the PALOMA-1/TRIO-18 trial (NCT00721409), an open-label phase II study in postmenopausal patients with ER+/HER2– MBC who had not received prior treatment for advanced disease (Finn et al., 2015). Initially, patients were enrolled into two cohorts: Cohort 1 was enrolled on the basis of ER+/HER2– status alone, whereas cohort 2 also required that cyclin D be amplified and/or there be a loss of p16. After an unplanned interim analysis of cohort 1, the statistical analysis plan for the primary outcome was amended to allow a combined analysis of both cohorts, and accrual to cohort 2 was stopped.

For patients treated with the combination of palbociclib and letrozole, the median progression-free survival (PFS) was 20.2 months (95% confidence interval [CI]: 13.8–27.5 months), compared with 10.2 months (95% CI: 5.7–12.6 months) in patients who received letrozole alone (hazard ratio [HR] = 0.488; 95% CI: 0.319–0.748; Finn et al., 2015).

The manufacturer has also initiated two phase III studies: the PALOMA-2 (NCT01740427) and PALOMA-3 (NCT01942135) trials. The PALOMA-2 trial, designed to confirm the findings of the PALOMA-1 trial, was a randomized (2:1), multicenter, double-blind study evaluating palbociclib in combination with letrozole vs. letrozole plus placebo as a first-line treatment for postmenopausal patients with ER+, HER2– advanced breast cancer (ClinicalTrials.gov, 2015a).

The PALOMA-3 trial was also a randomized (2:1), multicenter, double-blind phase III study evaluating palbociclib in combination with an alternative antiestrogen, fulvestrant (Faslodex), vs. fulvestrant plus placebo in patients with HR+, HER2– MBC. Unlike the PALOMA-2 trial, the PALOMA-3 trial enrolled patients whose disease had progressed after prior endocrine therapy. This study was stopped early due to demonstration of improved efficacy in the palbociclib arm, and presentation and publication of these results will likely occur in the near future (ClinicalTrials.gov, 2015b).

In addition to confirmatory studies to explore the role of palbociclib in combination with antiestrogen therapy and/or chemotherapy in MBC, trials are underway in other clinical settings and diseases, including early-stage breast cancer (ClinicalTrials.gov Identifier NCT01864746), non–small cell lung cancer (NCT0254490), glioblastoma (NCT01227434), head and neck cancer (NCT02101034), and multiple myeloma (NCT00555906).

## INDICATION

In February 2015, palbociclib received accelerated approval from the FDA in combination with letrozole for the treatment of postmenopausal women with ER+, HER2– advanced breast cancer as initial endocrine therapy for metastatic disease. This accelerated approval is based on the improved PFS observed in the PALOMA-1 trial, and continued approval of this indication will depend on verification and description of clinical benefit in the confirmatory ongoing PALOMA-2 trial.

## DOSING AND ADMINISTRATION

Palbociclib is taken orally once a day for 21 days followed by 7 days off to complete a 28-day cycle. It is administered in combination with oral letrozole at 2.5 mg daily throughout the 28-day cycle. The recommended starting dose for palbociclib is 125 mg; 100-mg and 75-mg capsules are also available.

Tables 1 and 2 outline dose modifications and toxicity management recommendations. Mild hepatic impairment and mild and moderate renal impairment had no effect on exposure of palbociclib. Palbociclib should be taken with food, as fasting conditions appeared to affect absorption in some patients. Doses should be taken at approximately the same time each day. Capsules should be swallowed whole and should not be opened, chewed, or crushed. If the patient vomits or misses a dose of palbociclib, an additional dose should not be taken that day. Complete blood cell count monitoring should be performed before palbociclib is started, every 2 weeks for the first two cycles, then prior to each cycle and as clinically indicated (Pfizer Inc., 2015).

**Table 1:**
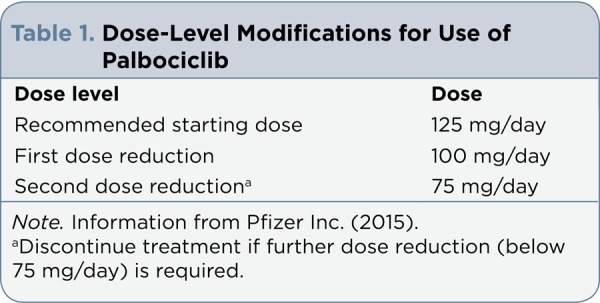
Dose-Level Modifications for Use of Palbociclib

**Table 2:**
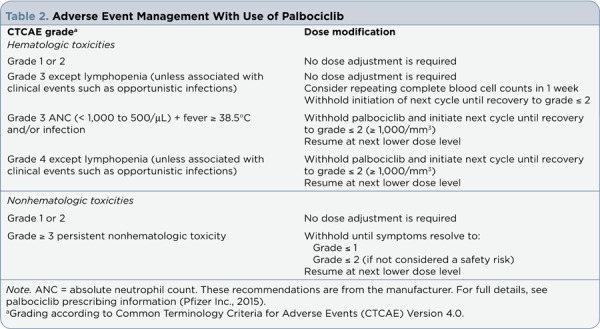
Adverse Event Management With Use of Palbociclib

## DRUG INTERACTIONS

CYP3A4 is an essential pathway in palbociclib metabolism. Concomitant use of strong CYP3A4 inhibitors and palbociclib should be avoided, as it may lead to an increase in palbociclib plasma exposure, resulting in greater adverse effects. If the combination cannot be avoided, the dose of palbociclib should be reduced to 75 mg. Patients should also avoid grapefruit and star fruit as well as their juices while taking palbociclib. Moderate and strong inducers of CYP3A4 should be avoided in combination with palbociclib, as they may reduce efficacy by decreasing the plasma exposure of palbociclib.

In vivo, palbociclib is a time-dependent inhibitor of CYP3A4; thus, coadministration of sensitive CYP3A4 substrates with narrow therapeutic windows may need to be reduced. Midazolam is one such medication; plasma exposure of midazolam was increased by 61% in healthy volunteers taking palbociclib (Pfizer Inc., 2015). A complete and accurate medication reconciliation should be performed before initiating any new medication or changing medications during treatment.

## ADVERSE EFFECTS

The most common grade 3/4 adverse reactions observed in the PALOMA-1 study with the combination of palbociclib and letrozole compared with letrozole alone were neutropenia (54% vs. 1%), leukopenia (19% vs. 0%), and fatigue (4% vs. 1%; Finn et al., 2015). Pulmonary embolism has been reported at a higher rate with the combination of palbociclib and letrozole (4% vs. < 1%). At least 10% of patients receiving the combination experienced thrombocytopenia, upper respiratory infections, stomatitis, decreased appetite, vomiting, asthenia, peripheral neuropathy, and epistaxis. However, most of these adverse events were grade 1/2.

Other frequently reported adverse reactions of any grade in patients receiving both palbociclib and letrozole include anemia, nausea, arthralgia, alopecia, diarrhea, and hot flashes. Patients should be counseled to report shortness of breath, fever, unusual bleeding, mucositis, or uncontrolled pain to their care provider.

## IMPLICATIONS FOR THE ADVANCED PRACTITIONER

There is a paucity of data on how best to manage patients with newly diagnosed HR+ metastatic breast cancer. Except in situations of large disease burden or rapid progression, endocrine-based therapies are typically the first-line treatment of choice. In addition to palbociclib plus letrozole, alternative early treatment options include fulvestrant (with or without an AI), everolimus [Afinitor] with an AI, an AI alone, and tamoxifen.

There has been a trend toward improved PFS using combination therapies vs. monotherapy with an AI, but to date, there have been no completed trials with a head-to-head comparison of dual-therapy regimens (fulvestrant and an AI vs. palbociclib and an AI vs. everolimus and an AI). It is not yet known whether there is a preferred sequence to using these new targeted and combination therapies.

Furthermore, triple therapy with antiestrogens, mTOR, and CDK4/6 inhibition may prove an even more effective treatment option than dual-therapy combinations, but this approach has not yet been studied. Therefore, clinicians should consider toxicities and quality-of-life issues for patients when making treatment decisions. Patients who would be a candidate for palbociclib are typically also candidates for fulvestrant; when fulvestrant is combined with an AI, there have been similar survival data and a more favorable toxicity profile, with no risk of myelosuppression (Mehta et al., 2012).

Clinicians must consider the impact of cost as well as potential toxicities when making treatment decisions. The retail cost of palbociclib approaches $12,000 dollars a month; this is much higher than, for example, anastrozole monotherapy, which retails in the United States for around $400 dollars a month (Lexi-Drugs, 2015). Patients’ out-of-pocket cost varies widely and is impacted by insurance benefits and limitations. There is a drug assistance program available through Pfizer for patients who qualify (PfizerRxPathways.com).

## SUMMARY

Palbociclib is a promising new agent that inhibits cell proliferation through restoration of Rb function. It is currently approved by the FDA in combination with letrozole for first-line therapy for newly diagnosed or relapsed ER+, HER2– MBC. As of April 2015, the limitation of its use in the first-line setting restricts its clinical utility, as there have been more significant toxicities relative to other regimens used in this setting.

Publication of further data will be beneficial in guiding the management of HR+ MBC, and ongoing research may lead to a broader indication for palbociclib in patients who have progressed on prior endocrine therapies.
